# A Geographic Information System Framework for the Management of Sensor Deployments

**DOI:** 10.3390/s100504281

**Published:** 2010-04-29

**Authors:** David J. Russomanno, Yury Tritenko

**Affiliations:** 1 Department of Electrical and Computer Engineering; the University of Memphis; Memphis, TN 38152, USA; 2 Woolpert, Incorporated; 215 East 32nd Street; Indianapolis, IN 46205, USA; E-Mail: yury_tritenko@yahoo.com

**Keywords:** GIS, service-oriented architecture, sensor ontology, sensor networks

## Abstract

A prototype Geographic Information System (GIS) framework has been developed to map, manage, and monitor sensors with respect to other geographic features, including land base and in-plant features. The GIS framework supports geographic placement and subsequent discovery, query, and tasking of sensors in a network-centric environment using Web services. The framework couples the GIS feature placement logic of sensors with an extensible ontology which captures the capabilities, properties, protocols, integrity constraints, and other parameters of interest for a large variety of sensor types. The approach is significant in that custom, GIS-based interfaces can be rapidly developed via the integration of sensors and sensor networks into applications without having detailed knowledge of the sensors’ underlying device drivers by leveraging service-oriented computing infrastructure within the GIS framework.

## 1. Introduction

In this era of ubiquitous sensors and sensor network deployments, the management and tasking of these sensors, including their associated metadata, sensed percepts, and capabilities, along with representing the underlying phenomenology, has become an enormous challenge. Geographic Information Systems (GIS) have wide-spread utility in a variety of domains for the management of complex data obtained from remote sensing, automated mapping and facilities management systems, and a myriad of other applications. Recently, some researchers are applying GIS to manage the placement of wired and wireless sensors and sensor networks across large, and often remote, geographic regions, as well as developing GIS interfaces to dynamically discover, query, and task sensors within a service-oriented architecture (SOA) [1].

The SOA and Web service standards, such as those endorsed by the World Wide Web Consortium (W3C), have broad industry acceptance and widespread use among software developers, particularly in business applications. However, there is relatively slow adoption by sensor designers to provide the infrastructure required for sensors to easily integrate within SOA applications, including GIS frameworks. Many ubiquitous sensing applications may not have been anticipated or programmed when the sensors were initially deployed; therefore, opportunistic, on-the-fly discovery, configuration, and tasking of sensors by software agents via GIS interfaces are critical services. Moreover, applications that utilize the composition of services with mashups [2], which combine algorithms and data from multiple sources, are driving the need for sensors that deliver access to data and embedded operations as standard Web services accessible via GIS applications.

Many sensor network systems require the application developer to have a detailed knowledge of the underlying software drivers to build applications. Many commercial sensor networking packages provide custom software to manage sensor node deployments for a few common types of sensors, such as those that can sense temperature, barometric pressure, light, and sound [3–7]. However, when building complex, enterprise-wide systems, end users have to deal with multiple possible applications for a given sensor or sensor node, with each having a specific device driver or management software. Enterprise-wide systems that are built using a variety of control software are difficult to manage [8]. Hence, one opportunity for advancing enterprise-wide sensor deployments is developing the capability to manage a group of heterogeneous sensors with multi-user access and extensive interoperability via GIS frameworks.

Applications built upon an enterprise-wide architecture must provide an interactive user interface for a wide variety of sensor types within a unified framework. Also, managing sensor deployments with respect to spatial information, including both land base and inside-of-plant features is critical. That is, the location of sensor deployments must be related to other objects of interest with respect to a land base map and other physical structures, such as buildings, to provide functionality for constructing complex queries, as is common in wide-spread GIS applications. Besides construction of geographical queries, interactive applications must also provide functionality for remote access to sensor data, as well as the ability to dynamically set sensor tasking parameters based on the end users’ high-level goals.

Several research and commercial projects have been developed to enhance sensor and sensor network interoperability with respect to geographic maps. One example is Microsoft’s SensorMap [9]. This internet-based application provides functionality for a user to indicate the placement of a sensor deployment on a geographic map, to query sensors by type, and to display sensed data. However, it does not appear that SensorMap provides functionality of a comprehensive GIS framework. With SensorMap, sensors are not related to spatial information of surrounding objects. In addition, the types of sensors available for deployment on the map are also limited. Additional related work includes the following: (i) sensor web-oriented GIS [10], which is a prototype application in which sensor web resources are regarding as data sources; (ii) the wireless sensor network application service platform (WASP) [11]; (iii) the geographic resources analysis support system (GRASS) [12], which is a large number of Web services built on top of GIS; and (iv) an expert system framework for live hazard monitoring and detection that has been integrated with GIS [13].

Other researchers use GIS platforms for sensor network deployments consisting of a few types of sensors with similar connection characteristics for monitoring parameters within a specific environment [14–16]. Nevertheless, these systems do not appear to have extensive support for networks of heterogeneous sensor types and do not appear to support various methods for placing, querying, and tasking the sensors with respect to other geographical features, such as land base and in-plant features.

A GIS framework has been prototyped in our laboratory to attempt to address some of the shortcomings of state-of-the art approaches to couple sensors with geographic maps and to also provide a GIS interface to support other ongoing sensor development projects. The prototype framework was developed using the ESRI ArcGIS [17,18] platform using a variety of commercial sensor networks, comprised of several sensor nodes, as well as custom sensors developed in our laboratory. The prototype leverages service-oriented computing principles to provide a layer of abstraction for query and tasking of the sensors and sensor nodes via the GIS environment.

Although a significant portion of the material in this journal paper appears in our work-in-progress conference publications [19,20], that work does not include all of the details and context of this paper. The remainder of the paper is organized as follows: Section 2 presents our GIS-centered architecture of sensors and sensor networks. Section 3 describes the implementation details of our prototype framework and Section 4 overviews user applications built and tested using the prototype. Section 5 offers conclusions and directions for further research.

## 2. GIS and Sensors System Architecture

**Figure 1.**
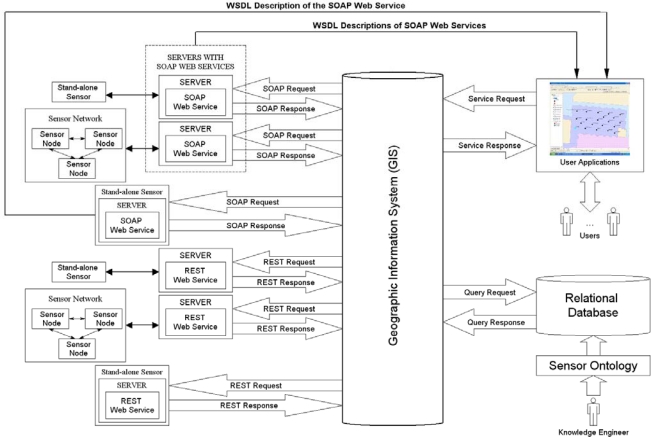
Prototype architecture for coupling sensor deployments with GIS.

A comprehensive architecture that couples sensors and networks within a mapping environment includes, but is not limited to, the following components: (i) sensing devices; (ii) sensor servers; (iii) GIS; (iv) database; (v) sensor ontology; and (vi) user applications. These components are shown in the prototype architecture in Figure 1.

Sensing devices are of two common types: stand-alone sensors and sensor nodes within a network, which are comprised of one of more sensing devices on the node. Stand-alone sensors are those that function autonomously and do not rely on other sensors or sensor nodes for communication or other functions. These devices do not have functionality for relaying sensed data to and from other sensors or sensor nodes; however, many stand-alone devices have a programmer’s interface (API) to task and retrieve data from the sensor. Stand-alone sensors can be integrated into a network, but are not regarded as nodes that form a traditional sensor network. Most of the APIs require detailed programming knowledge in a language, such as C, while a few APIs are beginning to provide higher-level programming abstractions, which are common in the service-oriented computing paradigm. Examples of these sensor types are imaging devices, including sophisticated visible, short-wave infrared, mid-wave infrared, long-wave infrared, and hyper-spectral imaging sensors, as well as stand-alone unattended ground sensors, such as gamma-ray, acoustic, and vibration sensors.

Sensor nodes are comprised of one or more sensing devices along with a supporting radio, which can transmit and receive data from other sensor nodes that comprise a sensor network. The sensor nodes form one or more paths back to a gateway or base station to collect the sensed data at a central server. Due to the low cost of most sensor nodes, single points of failure are common; therefore, the network can dynamically reconfigure the paths back to the base station. Sensor networks are an attractive alternative to stand-alone sensors when the nodes may need to be regarded as disposable or ‘place once and never access again’ as they are deployed over a large geographic territory.

A sensor server collects data from a stand-alone sensor or a sensor network. Some servers are also needed for the transfer of data to and from other entities or to other sensor servers or sensor nodes. Some sensor servers have been developed using service-oriented infrastructure so that high-level communications with other entities can occur for retrieval of sensor data or to relay commands to stand-alone sensors or sensor nodes.

Our GIS framework provides a high-level user interface for managing stand-alone sensors and sensor networks. A GIS development environment typically provides data visualization tools and other functionality to build interfaces to query and examine attribution of mapped objects. In the architecture shown in Figure 1, the GIS is a component that is coupled with a database system to organize and store data about sensor and sensor network deployments to support multi-user, enterprise-wide applications.

The database system coupled to the GIS framework includes a sensor ontology component as shown in Figure 1. The sensor ontology provides a machine-readable repository containing declarative knowledge about sensing devices, including metadata and other properties, as well as the sensing devices’ common capabilities and relationships to other sensors and underlying phenomenology [21–25]. Onto Sensor [21] is one example of some initial work in developing a sensor ontology. The ontology can be exported to a database segment as resource description framework (RDF) triples [26]. The database segment is then coupled to the GIS system to provide information to the user about the sensor types available for placement within the GIS environment. The sensor ontology development is ongoing, as new sensors are being designed and tested. The sensor ontology is administered by knowledge engineers, who must work with sensor and phenomenology experts to elicit the knowledge needed to populate the ontology.

The user applications are custom programs built for specific tasks and therefore depend upon the capabilities of the GIS. Custom interfaces developed within the GIS environment provide the functionality for users to select the types of sensors for placement on the map and to specify query and tasking parameters needed for the user’s application. The GIS interface also provides the primary communications portal from the user to the deployed sensors or sensor network.

Within the architecture shown in Figure 1, the transfer of data and commands between system components is performed using Web services hosted on the sensor servers, thus enabling service-oriented computing as the core paradigm for information exchange between sensors and users. For example, Figure 1 shows the use of the Simple Object Access Protocol (SOAP) [27] and Representational State Transfer (REST) [28] protocols for communication between the GIS and sensor servers. Both of these protocols have advantages and disadvantages, which are outside the scope of this paper. Communication via SOAP calls is performed using an additional messaging layer exposed as a Web Services Description Language (WSDL) [29] file. The REST protocol sends requests to Web services over Hypertext Transfer Protocol (HTTP) without an additional messaging layer. In both cases, responses from Web services are typically returned in the form of HyperText Markup Language (HTML) or Extensible Markup Language (XML) data.

A user’s request to a sensor or sensor network originates within the GIS environment and the specific protocol used for the underlying communication is determined based on attribution parameters of the sensing device. Requests are sent to the server and forwarded to the sensor through the Web service executing on the server. Responses from the sensing device are routed through the corresponding server to the GIS where it is displayed to the user. The architecture of Figure 1 shows that the Web service may reside on a separate server for a given stand-alone sensor or sensor network, or may be bundled as part of the sensor itself.

## 3. Architecture Prototype Implementation

A prototype GIS system has been implemented in our laboratory based upon the architecture shown in Figure 1. The prototype environment stand-alone sensors include an unattended ground sparse sensor detector (UGSSD) also referred to as a type of profile sensor [20], a Sony 1394 visible camera, and a FLIR A40 long-wave infrared camera. Also, sensor nodes from the Arch Rock Primer Pack [30] are included in the prototype environment. ESRI’s ArcGIS has been used as the GIS platform for the prototype system.

**Figure 2.**
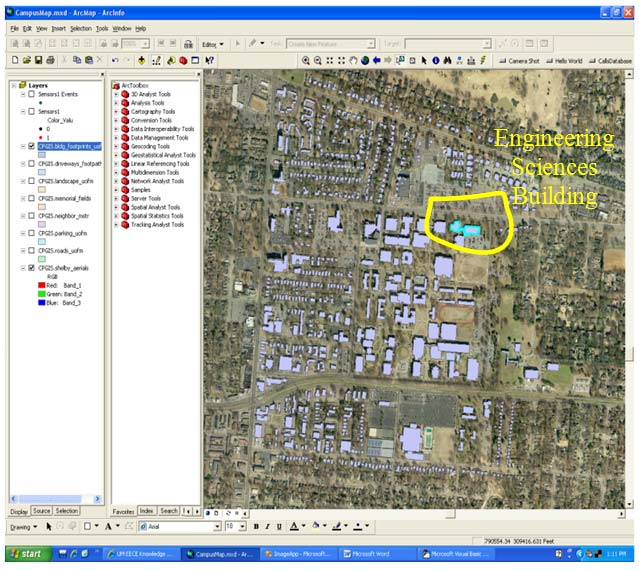
View of the U of M campus with building footprints and the Engineering Sciences Building selected (upper right) for deploying sensors.

In the prototype framework, a user can locate a geographical area of interest, such as a parking lot, and then can select a feature on the map, such as a utility pole or tower, and then can associate a sensor, such as a camera, to the utility pole in the parking lot upon placement of the sensor on the map. Figure 2 shows a graphic of the University of Memphis (U of M) campus with the engineering sciences building selected in the upper-right quadrant. Buildings are one type of geographic feature that can be associated to a sensor during placement on the map. Integration of more detailed geographic features than the U of M campus map shown in Figure 2 is readily supported by the framework and can be imported or created using the ArcGIS platform using standard GIS tools.

**Figure 3.**
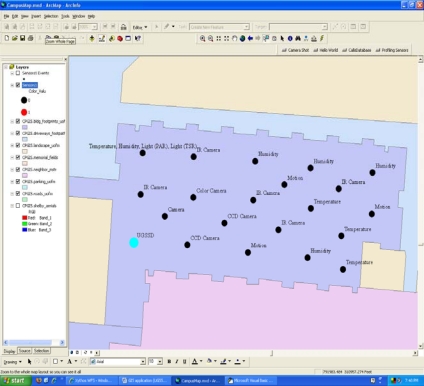
Inside plant view: Area of interest within the engineering sciences building with a heterogeneous sensor deployment. One UGSSD is highlighted in the lower left of the view.

Figure 3 is a zoomed-in view of an area within the engineering sciences building showing the placement of several sensors of various types. Inside-plant features, such as rooms or halls, or sub-areas within rooms, such as walls or ceilings, can be readily added to the framework using the ArcGIS platform or other GIS tools to provide more details for *in situ* references.

The sensor ontology contains knowledge about a variety of wired and wireless sensors, including the UGSSD, as well as other sensor types, such as infrared cameras, CCD cameras, motion detectors, temperature, light (PAR), light (TSR), humidity sensors, *etc*. Numerous sensor types are captured in a sensor ontology, which the GIS application queries as part of the sensor placement logic, to provide the user with a wide selection of sensor types and capabilities, as well as other parameters that may need to be specified to task the sensors.

The UGSSD sensor, which is one sensor in a family of profile sensors, was designed in our laboratory, and it is an example of a custom sensor that includes a Web services API. The UGSSD uses a collection of active, near-infrared sensing elements [31] that comprise the overall sensing device. The UGSSD is designed to detect and discriminate among different types of objects from profiles or silhouettes, such as humans, animals, and vehicles in an unattended ground sensor deployment [32–34]. The UGGSD can be tasked via configuration parameters to detect a variety of object types given various constraints, such as detect the number of occurrences of a specific object type crossing the sensor’s field of view during a specified time interval. For example, if ten humans crossed the sensor’s field of view during a two minute interval, the integer ten would be returned as the number of object occurrences. The UGSSD can also be tasked to send a notification e-mail when the object detection constraints have been satisfied. The communication with the UGSSD occurs via a Web service hosted on a sensor server executing Java and can be invoked via SOAP messages.

For the UGGSD, all sensor data and embedded operations, such as self-test, sensor sample rate, alarm thresholds, and other configuration parameters and functionality are being exposed as Web services using WSDL. Such a service-oriented architecture with Internet Protocol (IP) networking provides a framework for including the sensor in a variety of intelligent monitoring applications and deployment and interoperability within existing frameworks. For example, in a crude electronic fence application, a given deployment of the UGGSD sensor may be tasked to detect any object that it senses. This task is accomplished via the *detect_object* service call, which requires the client to specify the time interval for which the sensor should report a detect object event on placement of the sensor in the GIS environment.

**Figure 4.**
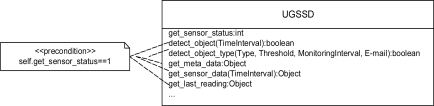
Example Web services for the UGSSD.

Increasingly sophisticated electronic fence applications that use the UGGSD may require the invocation of more detailed services, such as *detect_object_type*. This service requires the client to specify the object type to detect (for example, human, human_large_backpack, human_small_backpack, pickup truck, horse, sport utility vehicle, *etc*.), the threshold (e.g., an integer specifying the number of occurrences of the specified object type to detect during the specified time interval), the time interval for monitoring for the specified object, and an e-mail address to notify the recipient of the detection event, which satisfies the specified constraints upon placement of the sensor within GIS. Example Web services for the UGSSD are shown in Figure 4. Note that the majority of the service calls require the sensor to have completed a successful self test. This is represented as a UML constraint in Figure 4.

**Figure 5.**
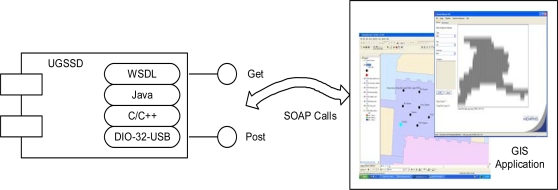
UGSSD component (UML deployment diagram): WSDL provides a Get and Post abstraction for the Web service in Java which insulates the application from the lower-level sensor API developed in C/C++. The GIS application maps the UGSSD sensor and can invoke a service to visualize the data captured from the sensor when a *detect_object* event is received.

The UGSSD provides a WSDL file that serves as a wrapper for the Java program that implements the Web service and subsequently invokes the lower-level sensor API developed in C/C++ as shown in the unified modeling language (UML) deployment diagram in Figure 5. The WDSL and the XML schema describe all types, methods, arguments, and responses of the services. The client application associates the abstracted types and structures specified by the WSDL file to the specific bindings required by the client’s host programming language. Figure 5 also shows a data visualization program that can be launched from the GIS environment to visualize data captured by the UGSSD, which in the figure below is data captured of a human silhouette. Using this approach, the application developer can use the programming development environment of choice when using the UGSSD in custom applications without the burden of knowing the lower-level sensor API, which is typically required when using sensors in custom applications. In the GIS application, SOAP calls are used to communicate with the UGSSD.

The sensor nodes from the Arch Rock Primer Pack include several sensing elements on the node for measuring temperature, humidity, total solar radiation (TSR), and photosynthetically active radiation (PAR). Each sensor node can serve as a relay device and can transfer data to and from other nodes via a radio channel or transfer data to and from the gateway server.

All sensing devices can be placed and visualized on the ArcGIS map. Maps in ArcGIS are sets of layers, tables, and raster datasets. Each sensor or sensor node in the prototype is represented on the map as a feature point. Client applications working within the ArcGIS environment have been developed to provide users with an interface for querying and tasking the sensors via Web services.

**Figure 6.**
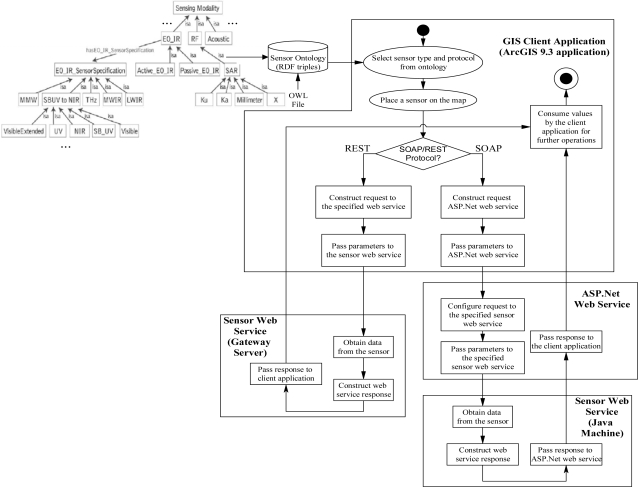
Web services command workflow associated with GIS sensor placement.

Due to some current limitations in the implementation of the prototype framework, the UGSSD Web services are not called directly from the ArcGIS client applications. The UGSSD’s Web services are called through ASP.Net Web service, which serves as a proxy service for calls to the UGSSD. Calls to ASP.Net must include a link to the WSDL file of the Web service specific to each deployment of the UGSSD. When a user specifies query or tasking parameters for a specific UGSSD deployment, the ArcGIS application, which is developed in Visual Basic for Applications (VBA), submits SOAP requests to the ASP.Net Web service and this call is transferred to the specified sensor’s Web service. After obtaining a SOAP response from the sensor’s Web service, it is passed back to the client application through the ASP.Net Web service as diagrammed in Figure 6.

The Arch Rock sensor nodes are queried and tasked using REST messages constructed in the ArcGIS user application. Communications with the gateway server also occur via the REST protocol. Messages can be transferred to and from the sensor nodes based upon the user’s request. The REST requests to the Arch Rock sensors’ Web service include the name or IP address of the gateway server hosting the Web service and the unique address of the specific sensor being queried or tasked. The request is sent directly to the sensors’ Web service where it is executed and the REST response, which is in the form of XML data, is sent back to the client application where it is presented to the user. This detailed Web services command workflow for invoking sensor services within the GIS prototype is also shown in Figure 6.

**Figure 7.**
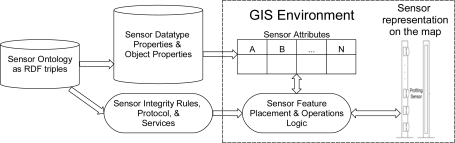
Interaction between sensor ontology and GIS environment.

The sensor ontology is exported to a database as RDF triples and is coupled to the GIS system (geodatabase) to provide users with information about sensor types available for placement within a given GIS environment. When the user places a new sensor on a map, the sensor ontology coupled to the GIS provides the types of sensors available for placement on the map, along with ontological information about the capabilities and services provided by the sensor. The sensor ontology also provides the user with sensor attributes, including those that can be controlled or set by the user within the GIS environment. These attribute values are populated from the sensor ontology to the attribute domains within the GIS environment as shown in Figure 7. Also, rules describing permissible values for features on the map are used to enforce data integrity constraints, for example, specification of the lower and upper wavelength, field of view, horizontal and vertical pixel count, frame rate, *etc*. for an imaging sensor.

## 4. Example GIS Applications

Two custom applications demonstrating the functionality of the prototype GIS framework will be overviewed next: (i) an interface for tasking deployments of the UGSSD; and (ii) an interface for tasking the Arch Rock sensor nodes. Both applications capture features selected on the map. When a given feature is selected, its attribution parameters are then read. In the case of the UGSSD, the attributes include the path to a WSDL file describing functionalities of Web services applied to the selected sensor. In the case of the Arch Rock sensor nodes, attributes include the name or IP address of the server where the Web services are hosted and a unique address of the sensor.

**Figure 8.**
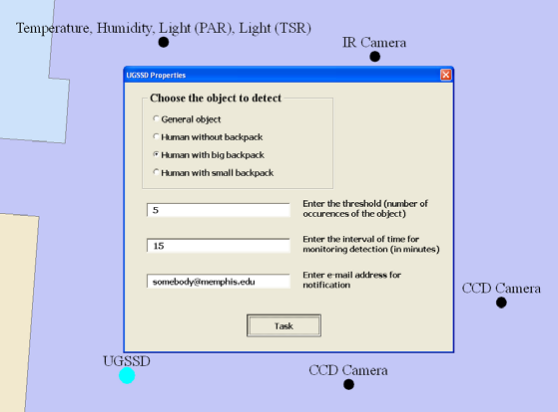
GIS interface for setting UGSSD tasking parameters. The interface requires four inputs to be specified by the user: (i) the type of object to detect; (ii) the threshold indicating the number of occurrences of the object to detect before a notification is sent; (iii) the time interval for monitoring for the detection of the specified occurrences of the object; and (iv) the e-mail address to which the notification message should be sent [20].

A UGSSD sensor is tasked after passing a list of parameters to the Web Service necessary for invoking its services. These parameters are specified interactively using a custom interface for the application built using VBA. For example, when the UGSSD sensor is selected, a user must specify the type of object to be detected by the sensor, a threshold indicating the number of occurrences of the object, the time interval to detect the number of occurrences specified, and the e-mail address of the recipient for the notification, as shown in Figure 8 [20].

The call to the Web service must include the path to its WSDL file. This parameter is used by the Web service to select the correct reference to the server interfaced to the sensor of interest. If the sensor detects an object satisfying the specified constraints then, the icon of the sensor on the map will change its state; for example, the icon will turn red if the constraints specified by the user are met.

**Figure 9.**
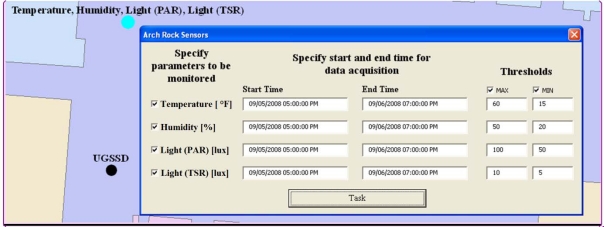
GIS interface for setting Arch Rock sensors tasking parameters. The interface requires the following inputs to be specified by the user: (i) parameters to be monitored by the sensor node; (ii) start time and end time for the data monitoring interval; and (iii) alarm thresholds for every parameter specified as maximum and minimum acceptable average values. The sensor state is represented by the symbology on the map.

When an Arch Rock sensor node is placed on the map or if it is selected for edit, the custom user interface prompts the user to specify the list of parameters required to build the underlying REST calls that are subsequently sent to the server. A user specifies the following: (i) environment parameters to be read from the sensor; (ii) precise dates indicating the time period the data from the sensor will be acquired; and (iii) an alarm threshold. The threshold indicates the maximum or minimum acceptable average value for every sensor parameter as shown in Figure 9. If an average value is over the maximum or under the minimum threshold, the application notifies the user by changing the corresponding attribution of this sensor and also modifying its symbol on the map.

## 5. Conclusions

A geographic information system (GIS) was used to build an interactive, multi-functional prototype development environment to manage sensors and sensor network deployments. The approach taken to build the prototype framework leverages a service-oriented computing infrastructure in which a programmer’s interface to the sensors has been abstracted using W3C standards, such as SOAP and REST. Web services are used for message exchange between users and sensors, including query and tasking functionality. A sensor ontology has been integrated into the GIS environment via an RDF triples database. Two user applications were demonstrated using the prototype for tasking stand-alone sensors, including an unattended ground sensor and a visible camera. Also, tasking many sensor nodes, which comprise a sensor network, was developed using service-oriented computing infrastructure.

Future development of the prototype framework includes the integration of several other sensor types into a comprehensive GIS environment coupled with an intelligent agent for dynamically discovering, querying, and tasking heterogeneous sensors to solve high-level goals. The GIS component will continue to provide the human user interface for managing and monitoring the deployment of heterogeneous sensors.

Alternative approaches to implementing the service-oriented computing architecture with Web services in our prototype framework are of interest. For example, the role of using Sensor Fabric [35,36] as a middleware infrastructure for sensor identification, discovery, access, and control within a GIS framework is an area worthy of additional research. With Sensor Fabric, a messaging bus is used to route control and management messages to publishers and subscribers, which include sensors, or other actors, such as a human or software services. In this scenario, the GIS application could be viewed as just another actor that subscribes to services provided by sensors and requests data or status from one or more sensors via a Fabric Manager, such that no knowledge of the detailed sensor device drivers or network topology are required. Nevertheless, the concepts and examples presented in this paper are of value even if alternative infrastructure to Web services is used to implement the service-oriented architecture.
